# Foreign Summary

**Published:** 1839-04-20

**Authors:** 


### FOREIGN SUMMARY.

*Case of Pericarditis with effusion. With remarks.* By Professor GRAVES.—The following case was so accurately noted, and the dissection so satisfactorily explained all the symptoms observed, that I have thought it worthy of publication. It is deserving of notice, that here, as in many other instances, increase of energy in the heart's action preceded the appearance of the more characteristic and essential signs of pericarditis, a fact seeming to denote that the disease often commences in the muscular substance of the heart, and from that extends to its investing membrane. Two years ago, Dr. Marsh, Mr. Lees, and I, saw a case strongly illustrative of this opinion. An athletic young gentleman contracted a very acute rheumatic fever from cold; the pulse was very high, the heat of skin excessive, and the pain, tenderness, redness, and swelling of the joints was of more than ordinary severity. He would not allow himself to be bled; we employed an antiphlogistic treatment, and were constantly on the watch to detect the first approach of pericarditis. One night Mr. Lees detected intermission of the pulse; this, in a few hours, was followed by increased strength of the heart's pulsations, and, finally, pain was felt. In many other instances I have observed irregular action of the heart to be the first signal of the approaching pericarditis: it is of importance to remember this, for it teaches us to attach more value to this symptom as a precursor of inflammation, and besides it proves that irregular and intermitting pulse may, in pericarditis, precede effusion, and do not necessarily arise from the impediment which the latter, when it takes place, must throw in the way of the heart's action.

Mary Kernan, ætat. 10, was admitted into the hospital yesterday, October 6th, in a state of collapse, moaning, sighing, and evidently suffering great distress from difficulty of breathing; the pulse could scarcely be detected; her extremities were cold, and considerable tenderness existed over the left side of the chest. Carbonate of ammonia, with calomel, and dry cupping to the painful parts, were ordered; and being this morning more at ease, and less in agony, she gives the following statements as the history of her illness.

Being placed in a draught of air, yesterday week, whilst lying in bed, was seized the following day with shiverings, vomiting, headach, pains in the loins, thighs, and legs, also a beating of the heart so strong as to make her imagine it would at last "thump" through her side, continuing for two days with slight intermissions in its violence; it was then accompanied with an acute, sharp, lancinating pain in the mammary region, extending to the neck and back, being particularly severe between the shoulder blades and the left arm as far as the elbow, and aggravated by motion, inspiring fully, or muscular effort of any description. Added to these complaints, there was difficuly of lying on the left side, with shortness of breath, and a hacking, distressing cough, without expectoration; this, however, she had had for many days previous, without any attendant pain or other untoward symptom. From the chest, the pain seemed to spread, or dart forward to the right side of the abdomen, and from thence over every part of the belly, occasioning more uneasiness than when confined to its primitive seat. Prior to coming in, some purgative medicines were given with slight relief. For several nights past, her sleep has been much disturbed, and she now lies on her right side, groaning frequently, and prostrated in strength, so as to be unable to raise herself in bed without assistance. She complains mostly of urgent thirst, a stuffing about the chest, and a "great weight, or heavy load on the heart," inability to lie on the left side, or sit up from an increase in the cough; pains in the mammary region, and palpitations of the heart; when pressure is made over this portion of the chest, much disquietude is produced.

Her countenance is bloated, œdematous, and pale; lips almost colourless; skin hot and dry; breathing rapid and laboured, 48 in the minute; pulse 120, small, feeble, varying in strength, and intermittent; tongue furred and clammy.

The left side of the chest to the eye appears fuller, of larger dimensions, and the muscles, as it were, puffed out; this is particularly obvious about the nipple; when measured, no inequality between the two sides can be discovered; percussion from an inch below the left clavicle, to the lower part of the cardiac region, also laterally over a space of several inches, is perfectly dull; this is likewise observable over the middle and inferior parts of the sternum, and to the right of this bone, whilst posteriorly over both scapulæ, as far as their spinous ridge, and below these bones, it is preternaturally clear. Respiration is exceedingly feeble over the dull parts, but free from rale, and elsewhere very loud. Impulse of heart cannot be felt; its action feeble, sounds indistinct below the mamma, becoming more audible towards the sternum, and can be heard in the epigastrium. No bruit can be detected. Abdomen full, tense, and much pained by pressure over the hepatic region.

Applicentur hirudines vi. regioni cordis et hypochondrio dextro. Hab. Pulv. Hydrarg. c. Creta gr. v. ter in die.

7th. Leeches were applied to the hepatic region alone; she expresses herself as somewhat relieved, and can now lie on the left side without being so much inconvenienced; slept better, and moaned comparatively little; pulse very irregular, is full and soft at one time for eight or ten beats, then diminishing in strength, it increases in frequency, to the rate of 120 or 130, gradually vanishes from beneath the finger, and ceases to be felt; the succeeding pulsations are full and distinct, not more than 88 or 90 in the minute. Respiration 48, still distressed; bowels open twice; tongue loaded and moist. Percussion over the parts noted above remains the same; on the clavicles of each side it is quite natural. Immediately above the left clavicle there is an evident fullness or swelling of the lower part of the neck, not visible on the right side; *and on coughing a tumour is brought into view, which disappears as soon as the paroxysm subsides.* Respiration in this part is perfectly distinct; a wheezing rale is audible in the lower portion of the left side. Heart's impulse and action the same; in the erect posture its sounds can scarcely be detected, but on lying down they are tolerable distinct.

Applicetur Vesicat. Epigastrio.
Repr. Pulv. Hydrarg. c. Creta.

8th. Was very restless the entire night, moaning frequently, and coughing constantly. Her countenance is less swollen; her breathing is more difficult; and she complains principally of the "stuffing and weight about the heart." Pulse remains of the same character, but is not so irregular.

No change has taken place in the phenomena either of the lungs or heart, except that the fullness in the lower part of the neck is more apparent, and the bronchitic rales more distinct in the inferior and middle portions of each lung. Abdomen not so tender, but still swollen; bowels purged.

Leeches were again ordered, and a further attempt made to bring the system under the influence of mercury, by inunction and the vapour of a mercurial candle.

9th. Breath is slightly mercurial; appears less affected in her breathing; the respirations continue rapid, 40 in the minute; no alteration in the character of the pulse.

There are now intense cooing and hissing rales in each lung posteriorly, but otherwise no change has taken place in the percussion or respiration. The cough is very troublesome, and attended with a frothy tenacious expectoration; pains increased, and palpitations induced by lying on the left side.

Repr. omnia ut heri præscripta.

Applicetur Vesicat. Hypochond. dextro.

10th. Prefers being in the erect posture, being more at ease, less oppressed, and in a great measure relieved "of the weight and load on her heart." Her countenance and aspect generally are improved, but her breathing remains frequent and laboured; *the pulse is regular,* 128 *in the minute, does not vary in strength, neither has an intermission ocurred during so many beats.* She is at present sitting up in the bed, and whilst in this posture the pulse was counted.

Percussion over the inferior portions of each lung posteriorly, the left in particular, has lost its tympanitic sound, but retains it at the superior parts. Heart's impulse is still imperceptible, its sounds are distinctly audible along the sternum.

Applicetur Vesicat. lateri sinistro.

Repr. alia.

11th. The pulse again varies in strength, intermits occasionally, and partakes of the description given on the 7th, is 120 in the minute, but she is now in the recumbent posture; passed the night, as heretofore, moaning and in a very restless manner; complains of the oppression about her heart being increased, and refers it to the lower part of the sternum and right side. There is considerable wheezing in the throat; on account of the blister, no examination of the chest could be made; pressure over the abdomen produces pain; it is swollen and dull all over when percussed.

Applicetur Vesicat. regioni Cordis.

Rep. ut. ulto.

12th. The phenomena remain as before, viz., fullness about the lower part of the left side of the neck, with pure and distinct respiration; healthy sound on percussion over each clavicle, with the natural vesicular murmur; one inch below this, better marked on the left side, extending over the middle and inferior parts of the sternum, anterior part of right side, and a portion of the lateral of the left, a perfectly dull sound is elicited by percussion; the respiration being almost null in the left, feeble but distinct in the right. A very clear sound on percussion in the superior parts posteriorly, with a mixture of bronchitic and crepitating rale in the inferior lobes, and loud respiration, free from rale, in the superior lobes. Heart's impulse and action the same. Pulse much weaker; respiration more frequent, 56 in the minute; breathing free; tongue loaded.

13th. Pulse almost imperceptible; breathing more laboured and distressed; lips of a livid hue. Died at 11 o'clock, P. M.

*Post Mortem fourteen Hours after Death.*—External appearance similar to that presented when alive; countenance puffed, pale, and œdematous; chest, *particularly left side, full and prominent,* and the abdomen distended and rounded. The same phenomena are afforded by percussion, as noted in the reports during life. The integuments of the chest, as also those of the abdomen, are watery. As soon as the knife pierced the cartilages of the left ribs, a gush of straw-coloured fluid took place, and when the sternum was raised, nothing but the pericardium could be seen, to such an extent was it distended, as to occupy the mesial line, extending from the diaphragm to within one inch of the fourchette of the sternum, and across to the right side. On removing it from the left cavity of the thorax, the lung was found much diminished in size, pushed upwards, and pressed against the spine and ribs. Having lost a great deal of its natural feel, and appearing like a lung compressed by a pleuritic effusion. The right lung was also affected in the same manner, but in a minor degree. Slight adhesions of recent formation existed between the left lung and pericardial sac, as also between the pulmonary and costal pleura, at the superior lobe of the right lung.

The pericardium itself is increased to at least three times its natural capacity; its exterior highly vascular, whilst its internal surface appears smooth, shining, and covered with a gelatinous kind of fluid, resembling the mucous coat of the stomach, or other portions of the intestinal canal. Its thickness is from three to five lines; but on inspecting the cut surfaces minutely, it is evident this increase is produced by the addition of a false membrane. On the superfices of this membrane are several patches of apparently coagulated lymph, stained of a purple or dark red colour; differing considerably in their dimensions, and situated in particular near the base of the heart, and that part of the sac in connexion with the posterior surface of this viscus; the larger of these, however, of an oblong shape, about two inches in length, and of a darker colour than the rest, is situated where the anterior part of the heart and pericardium are in contact. Besides these, there are innumerable depressions or pittings, capable of admitting the end of a probe on the lower and anterior part of this membrane, whilst near the base of the heart and the posterior part of its investing sac, this coating is separated into distinct patches, the serous covering of the pericardium being quite apparent underneath, and presenting its natural glistening appearance.

This false membrane can, with the greatest facility, be scraped off in solid pieces by the nail.

The entire surface of the heart is of a vermillion colour, and coated over with a most beautiful honey-comb, reticular kind of organized lymph, exceedingly fine, but perfectly adherent to the layer of serous membrane covering the heart at the apex.

Advancing upwards or nearer to the base, it is more condensed and compact, seemingly farther progressed in the process of organization, the shreds and interlacing fibres being increased in bulk.

From the quantity of this crimson-coloured network, at the commencement of the aorta and pulmonary artery, it is almost impossible to distinguish between them, so closely are they united together. The under surface of the auricular appendices, and that part of the heart they rest on, are the only portions which do not present to the same degree, and in a slight manner merely, the general aspect described.

Covered in this manner, and to such an extent as the anterior surface is, the posterior is trebly more so, and with a form of lymph more organized, denser and firmer, and from its exterior are three or four appendages, tough, closely adherent to, and evidently taking their origin from the surface of the coagulable lymph,

On the removal of a portion of this coating, the substance of the heart beneath presents a rosaceous hue; its size does not appear to be much altered, perhaps larger than natural. No examination of the interior. A quantity of the same coloured fluid escaped from the cavity of the abdomen on laying open its parietes; the liver did not appear increased in size, and its structure was perfectly healthy; bands of lymph passed between and connected together the visceral and parietal peritoneum, few and slight, and not connecting together the intestines themselves. The interior of the intestinal canal was not examined.

*Observations.*—In the preceding case the following points are worthy of notice:

1st. The great size of the tumour formed by the distended pericardium.

2dly. The protrusion of the left lung to a considerable extent above the clavicle, forming the tumefaction observed in that situation.

3dly. The tympanitic sound produced by the close application of the lung to certain parts of the *tense* pectoral parietes.

4thly. The varying states of the pulse, at one time intermitting and irregular; at another, free from these characters.

5thly. When admitted, copious effusion into the pericardium had already taken place, and yet the countenance was pale, and the lips colourless; there was no suffusion, no lividity, no venous turgescence whatever in the eyes, face, or lips; and yet her breathing was 48, and the pulse feeble, varying in strength and intermittent.

6thly. Although it is said in the report, that the left half of the chest did not measure more than the right, yet there was an evident dilatation of the former, exactly corresponding to the distended pericardium, which, pushing before it the flexible parietes, formed a well marked and evident prominence. This likewise rendered the parietes of the superior portions of the left side of the chest more tense than natural, an occurrence sure, for reasons well explained by Dr. Williams, to occasion increased resonance on percussion. *I am not aware that this consequence of pericarditis has before been described.*

*Dublin Journ. Med. Science.*

---

*On the Treatment of Scrofula.* By Mr. Benjamin Phillips.—Now, as to treatment, I would say, if a child is residing in a town, no matter whether in a large house or a small one, a change to the country is very desirable. The change of air, joined to the exercise in the open air which may there be taken every hour in the day, is unquestionably a very powerful means of preventing the development of the disease. With respect to the necessity for change of climate, I have much difficulty in pronouncing. Many circumstances would seem to warrant the opinion, that such changes may exercise a remarkable influence in the development of scrofula.

It is unquestionably a fact, that men apparently exempt from all scrofulous disposition or affection, are now and then attacked, when they quit a warm country to inhabit a cold one; and in these cases it is said the disease is more serious and more difficult to cure: the broad fact may be true; but we want to know, what has been the change in their habits as well as in the climate. Again, it is not easy to ascertain whether in youth a tendency to the disease was manifested. If a child be brought to you suffering from the disease, and you ask the parents whether they have suffered from a similar affection, you may be sure they will say no, and will vaunt the excellence of their constitution; they may say, probably, the nurse may have been tainted, or that their child has mixed a good deal with some children who had suffered. It is certain that animals transported from warm to cold climates ordinarily suffer from tubercles; but then it is difficult to estimate the influence of climate in producing the disease; their accustomed exercise in search of food is lost, and they are the denizens of a narrow space, boarded on three sides, so as to allow of a human minimum ventilation. On the other hand, it is certain that persons evidently scrofulous are frequently much benefited under the influence of warmer climates; in fact it seems to be by this circumstance that we are enabled to explain the amelioration which patients undergo during summer, whilst, during other portions of the year, it may resist every kind of treatment; and without seeking to deteriorate the merits of iodine, every one who has been accustomed to administer the different preparations of this medicine must have observed how comparatively inefficacious they are in the cold months; how decidedly advantageous is their exhibition during summer.

Before we proceed further, it is necessary to inquire whether there be any other particular circumstances under the influence of which remedial means present a better prospect of success. As to food, the course I am accustomed to pursue is, to afford my patients what is termed a generous diet, when there is no decided mesenteric affection to contra-indicate it; to give them a moderate quantity of animal food once a day, with well-cooked vegetables, and good bitter table beer, or wine and water. As to cleanliness, this must be carefully attended to, either by means of bathing or sponging; the surface of the body should be daily abluted and rubbed for some minutes, until thoroughly dry, and the capillary circulation of the surface be stimulated by means of warm towels. A most important element in the treatment, and one which cannot be too much insisted on, is exercise; but it must not be that kind of gentle exercise which invalid children left to themselves are too apt to take, but such as will largely employ the muscular system; they should be taken out twice or thrice daily in winter, if possible; and in summer they should be very little in the house during the day. It is necessary that games should be provided for them, so as to secure active motion for as long a time as the patient can bear it without fatigue. Indeed, I hold this to be one of the most decided preventives of this disease. I am so strongly impressed with the value of this agent, that I willingly subscribe to an opinion I have somewhere seen maintained, that by the well-directed employment of strong muscular exercise, many cases of this disease, where even tumours are found in the neck, may be cured. I hold it, therefore, to be necessary, that the several means to which I have now alluded should form the groundwork of our treatment of this disease, to which should usually be added the exhibition of certain medicinal substances.

Various systems have been greatly eulogized by their respective inventors. Many of them have long been consigned to oblivion, and probably some of those still retained, might, without loss, share a similar fate. I have fairly tried four of these systems, and I shall lay before you the results: those which I have employed are the antiphlogistic; in which I include the use of purgatives, emetics, blood-letting, and counter-irritation, the treatment by the various preparations of iodine, the alkaline treatment, and the mercurial.

Purgatives are unquestionably useful when conjoined with the general means to which I have alluded, and will frequently very manifestly modify, if not cure the disease, and they are especially valuable as an adjunct to the other modes of treatment: they are particularly useful when given during those periodical interruptions which are necessary in the treatment by iodine. How they exercise this beneficial influence is not so easy to explain—whether by exciting the action of the stomach and intestines, by procuring serous evacuations, or by other means; so much, however, is certain, that they are often of great use, and especially as an accessory means of treatment. The impression produced upon my mind is, that those purgatives are most beneficial which procure fluid evacuations, those into which saline substances enter.

My own experience does not enable me to recommend emetics with so much confidence as seems to have been felt by Bell, Smyth, Bordeu, Kortum, or Dussassoir. Undoubtedly, scrofulous children very commonly present a furred tongue, which is often not cleaned by the use of purgatives; such a case is often much benefited by one or two emetics; but beyond this my belief in their efficacy does not extend. I have never known the frequent use of emetics to be succeeded by any greater amount of amelioration than is usually experienced from the exhibition of two or three.

I have never known more than a passing relief to result from *blood-letting*; and this might naturally be expected, if it be true (and there is every reason to believe it is) that in scrofula the serum largely predominates in the blood. The abstraction of any quantity of blood must necessarily lessen that proportion, and as necessarily increase the evil which it is intended to remedy. The action of purgatives, when they produce watery stools, is the opposite of that. They occasion the exhalation of a considerable quantity of serous fluids upon the mucous surface of the intestines; and by so much lessen the preponderance of the serum in the blood.

In the last and the preceding centuries, it was currently believed that we possessed a power of neutralizing the condition upon which the tendency to abnormal deposits depended; and that power was supposed to exist in the *old subcarbonate of potash, or salt of tartar*. Levret believed that it was capable of rendering all deposits fluid, and that in this condition they might be absorbed or evacuated. Although, in the present day, we are satisfied that such virtues are not found in this substance, yet a sort of vague, undefined impression seems to exist, that it is not wholly useless even in scrofula. The Elixir of Peyrilhé, used in France up to the present day, is a mixture of this substance with infusion and tincture of gentian. In the *Collectanea Hauniensis* is a case of rickets, which appears to have been successfully treated by this medicine. Internally, I have given this medicine in small and large doses, in almost every form of scrofula, whether affecting the glandular, the mucous, the osseous, or the fibrous tissue; and I am unable to point out any case in which any small amount of relief which may have been obtained during its use could be fairly referred to this medicine.

In 1784, Crawford proposed as a remedy the *muriate of baryta*, and it was well received; it was very generally used through the greater part of Europe. Suddenly, two very opposite opinions were propagated with regard to it: one, that it was a useless addition to the materia medica; the other, that it was an agent of great energy, and that its exhibition, unless very guardedly, was not without considerable danger. These opinions were no sooner published than its use was abandoned, without, as it appears to me, any fair trial, in every country of Europe except

Austria. The Austrians were satisfied that in this medicine they possessed a very valuable agent in the cure of scrofula, and my own experience has convinced me that they were right, and that with the exception of iodine, no medicine seems to exert a more decided influence over scrofula than the muriate of baryta. It usually increases the appetite to about as great an extent as we see in children who are taking moderate doses of iodine; it increases all the secretions, and sometimes, like some of the forms of iodine, produces diarrhœa.

In twelve cases where it was exhibited in the dose of, at first one-third and afterwards half a grain, three times a day, no unpleasant symptom was developed. Eight were materially benefited by its employment. The general health improved sensibly, and the enlargement of the glands was very considerably lessened. In the other four cases no sensible influence was exerted over the disease. At the same time, however, that I am fully sensible of the valuable character of this medicine, I am bound to admit that its curative effects are less powerful—less certain—than those of iodine, and, therefore, for some time I have ceased to employ it. Several times I have proposed to use it alternately with iodine, or, when it has been necessary, to intermit the employment of the latter; but I have not yet carried this intention into effect.

Iodine, in its various forms, I have used extensively; and I have had very ample opportunities of estimating the relative merits of the different preparations of this substance. I have administered it in the form of tincture mixed with water, and also associated with the iodide of potassium. I have exhibited the iodides of iron, lead, sulphur, and arsenic. I have employed it externally, in the form of ointment, lotion, and bath; and as a broad or wholesale result, I may state shortly, that at present I rarely use internally any other form than the iodide of iron, and that the dose does not exceed, in any case, three grains, three times daily. I do not object to the tincture, because, as is alleged, the iodine is thrown down in a pure state when dropped into water, and so applied to the mucous membrane of the fauces and stomach, and is apt to create irritation there; but because I found that diarrhœa was an occasional consequence of its use—that it was inconvenient to trust the persons ordinarily found about patients to administer it—or because, when mixed in considerable quantity, a certain portion is precipitated, and because I found, in the ioduret of iron, a more valuable and certain remedy. I am quite ready to admit that many of these inconveniences were lessened by the combination of iodine with the iodide of potassium, suggested by M. Lugul; but the objection I found to attach to this form of administering the medicine, was the bulk of the vehicle, which very frequently disordered the stomach; and when I have lessened it, I have usually seen disorders of the stomach and intestines as a consequence. And in several cases, although the doses have always been moderate, the poisonous effects of this medicine have been developed; and I have no doubt that these effects would have been more frequently seen, had we not from time to time interrupted the treatment.

Internally administered, I have had no reason to speak strongly in favor of the iodides of mercury, lead, and arsenic. The first and last are unquestionably energetic preparations, but I think them better adapted to certain obstinate diseases of the skin than to scrofulous tumours, and even externally, except in a very dilute form, when they may unquestionably second the internal administration of the medicine: if the quantity of biniodide of mercury exceed ten grains to the ounce of lard, the irritation excited upon the part where it is rubbed will be such as to prevent our continuing it. The preparation of lead, in the proportion of a drachm to the ounce of lard, rarely excites similar irritation.

I have a register of 172 cases in which I have exhibited the iodide of iron. The minimum dose has been a grain twice a day, the maximum three grains three times a day. Of these cases, only twice was it necessary to intermit the use of the medicine for a few days: in one of these it excited ptyalism; it was laid aside for a fortnight, again resumed, and again produced ptyalism. Since that period, and within the last three months, the same patient, on her return from Margate, has been taking the medicine with the most decided good effects, and without ptyalism. In the other case diarrhœa supervened; the medicine was withheld for ten days, was then resumed, continued for several weeks, and without any derangement of the bowels. About once a week an aperient or purgative is given, which decidedly assists the treatment, but no other suspension of the medicine occurs. Where scrofulous ulcerations occur, whether as a consequence of abscess or from other cause, I am accustomed to employ, with the very best effect, a lotion containing three or four grains of this preparation to the ounce of distilled water.

In the employment of iodine or the iodide externally, one fact cannot escape a superficial observer, and that is the rapid change which follows the application. For a few days this diminution is very striking, but it is not long continued, and after a fortnight or three weeks the tumour appears stationary. Then is the time for resorting to a new form, which must be employed for a similar period, and must then give place to a third. But although these external applications will occasion a marked diminution of such tumours, they hardly ever completely disperse them; and when applied alone, without a concurrent internal administration of some preparation of the medicine, their effects are much less decided.

When such tumours are extremely indolent, the ointment may be rubbed upon the part without fear of injury; but if they be the seat of irritation, it is very likely to be increased by friction. In consequence of this circumstance, I usually recommend it to be applied to the part spread on lint. It is thus kept in contact with the surface for a much longer time, the irritation consequent upon rubbing is avoided, and the good effects of the medicine are more decidedly marked than by any other mode of application.

Many authors speak of the great or partial emaciation consequent upon the use of iodine. Jahn describes cases in which the emaciation was general. Coindet has referred to a diminution of the mammæ. Hufeland also gives three examples of it. Others have referred to the testicle as suffering in a similar way. And these isolated cases, which may or may not have succeeded to the use of iodine, are erected into a general law. Now, in my own experience, so far from emaciation of the whole or a part of the body being essential to the therapeutical action of this medicine, when prudently administered, one of the earliest symptoms observed is a remarkable increase of appetite, and a corresponding increase in the bulk of the body. I have watched its effects with great care, and I have not known a single case in which either the whole or even a part of the natural structures of the body have undergone any such change.

My experience of iodine in the form of baths is inconsiderable: such as it is, it leaves no desire on my mind to extend it. In two cases a considerable and troublesome eruption on the skin was produced; in three cases vertigo, with a suffused countenance, was occasioned, which was not dissipated for many hours, and no sensible good effect was produced on the tumours. These circumstances, added to the costly nature of the remedy, have deterred me from prosecuting further this mode of treatment. I know that this opinion is in opposition to that of Lugol, who is satisfied that the cure of these diseases is much accelerated by the conjoint use of baths and internal remedies; but any one who reads the cases given by Lugol cannot fail to recognise the same effects which I have described, though with less intensity. However, Baudelocque has come to a conclusion not very different from my own. Still he points out a remarkable effect which he has observed upon suppurating surfaces: he has always seen the suppuration much diminished, and the surface contracted; so that for some days much less linen was required for dressing the patients; but this effect does not seem to have been permanent.

Relying upon the encomiums of Hufeland, Charmeil, and others, I resorted to the use of *black sulphuret of mercury* in the treatment of this disease; but, whether associated with hemlock, magnesia, or ipecacuanha, I found no sufficient reason to induce me to employ it generally. I do not deny that the disease is often gradually but slowly ameliorated during the administration of this medicine; and I have never known any unpleasant effects—such as salivation—to result from its use; on the contrary, the tongue and the evacuations have improved under it, but with much less certainty and a much greater loss of time than under the influence of iodine. I prefer it to the common mercurial remedy employed in such cases—calomel and rhubarb—because, with the exception of the amount of good produced by evacuating the bowels, I have never seen any decided antiscrofulous virtue manifested by it.

Though under the influence of those remedies which we have just been considering, a patient's general health may be very decidedly improved—though glandular tumours may lessen—and even where suppuration has taken place, and the integuments over it have become thinned, they may be dissipated—yet where scrofulous matter has been deposited in its cheese-like form, neither iodine nor any other remedy which we know has power to procure its absorption; where it is deposited there it must remain; a point around which irritation is easily kept up, and about which, sooner or later, suppuration will take place; the abscess will either break, or art will interpose to facilitate this result by puncture, and it may thus be eliminated from the system. True it is, however, that the disposition to deposit this matter may be neutralized, and that all the more fluid portions of matter so deposited may be absorbed, and that, after death, a mass of cretaceous matter will be found to occupy its place. But in a large number of cases, spite of the most prudent treatment, the local disease will end in abscess; for instance, out of 89 cases, 33 presented this termination. When this is inevitable, it is unquestionable that we ought to anticipate by puncture, or other means, that gradual thinning of the tissues to which nature resorts in accomplishing the object; but it is not an easy matter to determine whether or not a scrofulous abscess will advance or retire; we may see the integuments so thinned that only the cuticle would seem to prevent its emptying itself, and yet it will retire—the whole of its contents will be absorbed. It must, however, be borne in mind that such abscesses are usually found to occupy the cellular tissue, and sometimes a lymphatic vessel, where no gland exists; in those cases where the abscess surrounds a gland where the product deposited in the substance of the gland is a constant source of irritation, the onward progress of the disease is more probable. It would, of course, be desirable that not only the thin sero-purulent matter which is usually contained in such abscesses, but also the scrofulous product, should be evacuated before the thinning has proceeded far, and the violet colour of the integuments is displayed; but this is a desideratum not easily accomplished. If the product have not undergone softening, often no evacuation of the matter will take place; if it have, a slight oozing, bringing away from day to day small portions of this matter, will be the course of evacuation, and often many months will elapse before the gland and its contents shall have been evacuated; and at the end of that time an unsightly cicatrix will be the consequence. This result is accomplished in the following way: one or two small openings in the thin violet-colour integuments are the channels through which the matter is discharged. A more or less extended cavity exists under, produced by the breaking down of the gland and its surrounding cellular tissue. When the whole of this structure is broken down and evacuated, this surface presents granulations which have a tendency to skin over without adhering at all, or on other occasions only partially to the superjacent thinned integuments. The consequence of this is an irregular puckered surface; and when, as is often the case, the subjacent tissue becomes adherent to the deeper-seated organs, the deformity is increased by a pitting. To prevent this aggravation, two modes may be resorted to. When the time for procuring the evacuation of such a tumour has arrived—when the integuments have become much thinned—the best mode of opening it is by applying the Vienna caustic paste to the part, taking care that the paste shall include the whole of the thinned structure. A fair and sufficient opening will thus be made; the evacuation will be more speedy, the remaining tissues will be healthy, and the cicatrix will be comparatively trifling. If, however, this have been neglected, or another course pursued—if the discharge be going on from one or more small points—if the integuments over the parts be very thin—then with scissors excise the whole of the violet integuments, and you may hope to lessen the deformity which would otherwise succeed to the disease. But much valuable time would probably be lost in the endeavour to heal the sinuses connected with the cavity; the various forms of iodine, in a more or less concentrated state, would have been applied to them, and the patient subjected to much suffering. And here I may state that after ample experience of such applications to these sinuses, I am decidedly of opinion that they occasion more pain and are much less efficacious than the nitrate of silver.—*London Medical Gazette.*

---

*Drs. Crampton and Marsh.*—Her Majesty has been pleased to grant the dignity of Baronet of the United Kingdom to Dr. Crampton, the surgeon-general in Ireland. Dr. H. Marsh, physician in ordinary to her majesty, in Ireland, has had the same honor conferred upon him.—*Ib.*

---

*On the Cicatrization of Tendons.* By M. Bouvier.—M Bouvier has been lately engaged in making experiments on this subject; and he exhibited some of the results at a recent meeting of the French Academy. His experiments went to ascertain—1, how far the divided ends of a tendon may be separated from one another, immediately after their division, so as not to prevent their reunion by any intermediate tissue; 2, in what degree the new tissue approaches to that of the true tendinous matter. The tendinous cicatrices presented to the Academy were taken—1, from a horse, two months before it was killed, and consisted of a perforating tendon of one of the anterior extremities; 2, from a dog, which was killed six months after the division of the extensor tendon of the foot. Each of these cicatrices was about two inches and a half in length. That of the horse consisted of a grayish, compact tissue, consisting of irregular fibres not very conspicuous, contrasting with the pearly appearance of the true tendon. The cicatrix of the dog consisted of longitudinal fibres, more delicate than those of the tendon, and arranged differently, being also very distinct from these.—*Brit. and For. Med. Rev. from Bulletin de l'Academie de Médecine.* No. xvi. 1838.

---

*Nearly complete Obliteration of the Pulmonary Artery.*—Dr. J. A. Power presented an example of this rare lesion, taken from the body of a young girl, who had been born with morbus ceruleus, and who died from an attack of bronchitis, at the age of twelve years.

The foramen ovale was not completely closed: the orifice of the pulmonary artery presented the appearance of a small papilla, through which it was barely possible to pass a fine probe; the tricuspid valves were puckered and contracted, and must have admitted of a free regurgitation into the right auricle, which was greatly enlarged and thickened; the musculi pectinati were highly developed; the right ventricle was also hypertrophied and dilated; the left auricle and ventricle were much reduced in size, and the termination of the right ventricle formed the apex of the heart. In this case the pulmonary circulation must have been carried on by the bronchial arteries.—*Dublin Journal.*

---

*Cancer of the Lung and Heart.*—Dr. Law presented a drawing, exhibiting extensive cancerous disease of the left lung; numerous tumours, of various sizes, were found imbedded in the substance of the organ; some having a consistence similar to that of brain, others were hard and cartilaginous; in some of the latter, a milky fluid existed in the centre; and they presented a fibrous structure, the fibres occasionally presenting a concentric arrangement; a mass of soft encephaloid matter was diffused through the centre of the lung; several tumours, likewise of a cancerous nature, existed between the base of the lung and the diaphragm; and one was noticed upon the anterior surface of the heart, and another imbedded in its muscular structure; softened tubercles existed in the liver, and one of the costal cartilages presented a tumour of the same fibrous character as those observed in the lung. *Ib.*

---

*English Medical Society at Paris.*—It may be interesting to the profession to know that an English medical society has been established in Paris, since the commencement of last session, and that it now includes between ninety and a hundred members. Evening meetings are held weekly, at which papers are read, and discussions take place: these meetings are peculiarly interesting, as men from London, Edinburgh, Dublin, and different parts of America, join in the proceedings, as well as some of the "internes" and "externes" of the Parisian hospitals.—*London Med. Gaz.*

